# A New Endoscopic Device to Facilitate APC Treatment: First Multicenter Clinical Experience

**DOI:** 10.1055/a-2934-1208

**Published:** 2026-08-20

**Authors:** Julius Mueller, Armin Kuellmer, Arthur Schmidt, Moritz Schiemer, Robert Thimme, Benjamin Walter

**Affiliations:** 1 Department of Medicine II, Medical CenterFaculty of Medicine, University Hospital of FreiburgFreiburgBWGermany; 2 Department of Gastroenterology, Hepatology and Endocrinology 15000 Robert Bosch HospitalStuttgartBaden-WürttembergGermany; 3 Department of Internal Medicine I, Gastroenterology 27197 Ulm University HospitalUlmBWGermany

**Keywords:** endoscopy upper GI tract, reflux disease, Barrett’s and adenocarcinoma, non-variceal bleeding, RFA and ablative methods, endoscopy lower GI tract, lower GI bleeding

## Abstract

**Background and Study Aims** Argon plasma coagulation (APC) is an established ablation technique, but limited by tangential probe orientation. ArgoCap was developed to allow precise, safe, and reproducible APC through visualizing the argon beam, shielding the probe, maintaining a defined distance, and enabling hybrid APC procedures. This study aimed to evaluate the clinical feasibility and safety of ArgoCap across multiple indications.

**Patients and Methods** Between January 2022 and May 2025, 54 APC procedures using the ArgoCap were performed at three expert centers. Indications included Barrett’s mucosa, ectopic gastric mucosa, gastric angioectasia, gastric antral vascular ectasia, radiation proctitis, and other indications. Primary endpoint was technical success, defined as access to the lesions and macroscopically complete ablation. Secondary endpoints included technical complications, adverse events, and procedural time.

**Results** The majority of lesions were located in the esophagus (70%), followed by stomach (19%) and rectum (11%). Median lesion size was 13 mm (range 2–240 mm). Median ablation and procedure times were 8 min (2–15 min) and 25 min (8–75 min), respectively. Technical success was achieved in 47 cases (87%); subtotal ablation (>90% of the lesion) was possible in 52 cases (96%). Technical difficulties occurred in 1 case (2%); primary complication (ulceration with subsequent bleeding) was observed in 1 case (2%).

**Conclusions** The ArgoCap enables easy and precise APC in the upper and lower gastrointestinal tract across multiple indications. It facilitates lesion access and visualization, reduces unintended mucosal contact, and allows hybrid APC procedures.

## Introduction

Argon plasma coagulation (APC) is an effective and well-established thermal ablation technique in endoscopy for years. It is the first-line therapy for angiodysplasia and radiation proctitis 1 2 3 and is also routinely used in the endoscopic treatment of Barrett’s mucosa and gastric antral vascular ectasia (GAVE). 4 5 APC is also an effective therapeutic option for the ablation of ectopic gastric mucosa (gastric inlet patch) in refractory reflux disease. 6 Conventional APC involves advancing the argon probe through the working channel of the endoscope and guiding it to the target site. Due to the forward orientation of the probe parallel to the scope in the lumen and gastrointestinal peristaltic motion, the endoscopic view of the target tissue may be limited. This makes targeted ablation more difficult, and unintended direct and straight mucosal contact or contact to healthy tissue is sometimes unavoidable. Complications associated with APC therapy are usually caused by the thermal energy applied to the tissue and can lead to esophageal strictures and, less commonly, perforations. 7 8 Accordingly, the duration and area of ablation should be kept to a necessary minimum. To overcome these limitations, the ArgoCap was introduced in 2023. The device is designed to simplify conventional endoscopic APC therapy. It allows continuous visualization of the plasma beam by maintaining the APC probe at an angled position in front of the endoscope, thereby facilitating stable probe guidance and secure fixation during treatment. In addition, it protects the probe from direct tissue contact and is intended to enhance endoscopic handling. The feasibility and safety of the device were demonstrated in ex vivo studies and in vivo experiments. 9 Clinical data on its use are not yet available. This multicenter observational study aimed to assess the handling characteristics and technical success of the ArgoCap and, as a secondary objective, to evaluate procedure-related complications associated with its application in the upper and lower gastrointestinal tract for various indications.

## Patients/Material and Methods

In this retrospective observational study conducted at three German tertiary endoscopy centers (University Hospital Ulm, Robert Bosch Hospital Stuttgart, and University Hospital Freiburg), all patients who underwent an endoscopic procedure involving argon plasma coagulation using the ArgoCap in the upper or lower gastrointestinal tract between January 2022 and May 2025 were included. Approval has been granted by the Ethics Committee of the University Medical Center Freiburg. Suitable patients were informed prior to the endoscopic procedures about the use of ArgoCap and the use of their data for research purposes. Following the procedures, the predefined parameters were documented internally at the centers. The data were collected and analyzed in anonymized form. The device was applied across different indications, including Barrett’s mucosa, ectopic gastric mucosa, GAVE, angiodysplasia, and radiation-associated proctitis. Primary endpoint was technical success, defined as endoscopic access to the target lesion and macroscopically complete ablation. Secondary endpoints included technical complications, procedural time, and procedure-related adverse events. Mild adverse events were defined as events not requiring medical or repeated endoscopic intervention and not prolonging hospital admission, whereas moderate adverse events required medical or repeated endoscopic intervention and/or prolonging hospital admission. Severe adverse events were defined as requiring surgical therapy and/or being potentially life threatening.

### Equipment and Settings

**Fig. 1:**
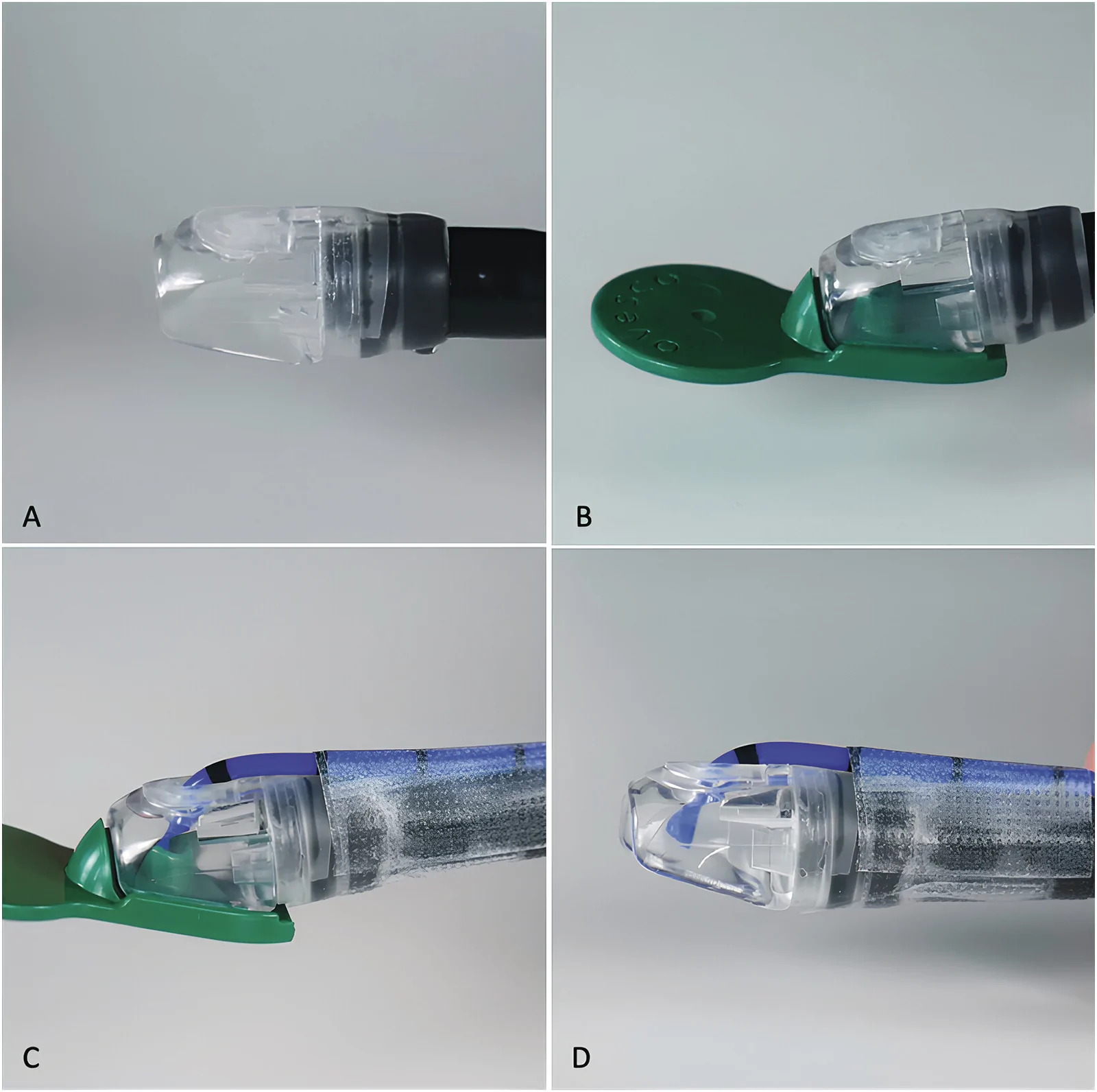
Assembly of ArgoCap. ( **A** ) Place ArgoCap onto endoscope, ( **B** ) Mount spacer, ( **C** ) Insert APC probe until it stops and secure with tape, ( **D** ) Remove spacer.

The ArgoCap (Ovesco, Tübingen, Germany) is a transparent plastic cap mounted on the distal tip of the endoscope with a flexible silicone grommet ( Fig. 1 ). It is compatible with all commercially available therapeutic gastroscopes (outer diameter 9.2–10.5 mm). In addition to the cap, the system comprises a spacer for probe adjustment, a plastic cover for the endoscope, and pre-cut adhesive strips for additional fixation of the probe and cap.

Conventional axial APC probes (diameter 2.3 mm) are inserted externally alongside the endoscope into a designated channel of the cap, where they are deflected in a predefined angle. The probe is fixed at a defined distance from the cap opening. The cap design also permits the introduction of an injection needle through the working channel of the endoscope, thereby enabling submucosal injection before APC treatment (hybrid APC). All procedures were performed using therapeutic video gastroscopes (Olympus GIF-190 or Olympus GIF-1500; Olympus, Tokyo, Japan). Electrosurgical generators (ERBE VIO 2 or ERBE VIO 3; Erbe, Tübingen, Germany) and identical axial APC probes (FiAPC 1500 A; Erbe, Tübingen, Germany) were used. Power output and APC settings were individually adjusted by the endoscopist’s discretion according to indication and anatomic location.

### Statistical Analysis

Continuous variables are presented as means ± standard deviation, while categorical variables are expressed as frequencies and percentages (in parentheses), unless stated otherwise. For categorical variables, differences were determined using χ ^2^ or Fisher’s Exact tests as appropriate. *P* values < 0.05 were considered being significant. Data collection and statistical analyses were conducted using Microsoft Excel 2021 or Mac (Version 16.103.4; Microsoft, Redmond, USA) and IBM SPSS Statistics for Windows (Version 31; IBM, Armonk, USA).

## Results

**Fig. 2:**
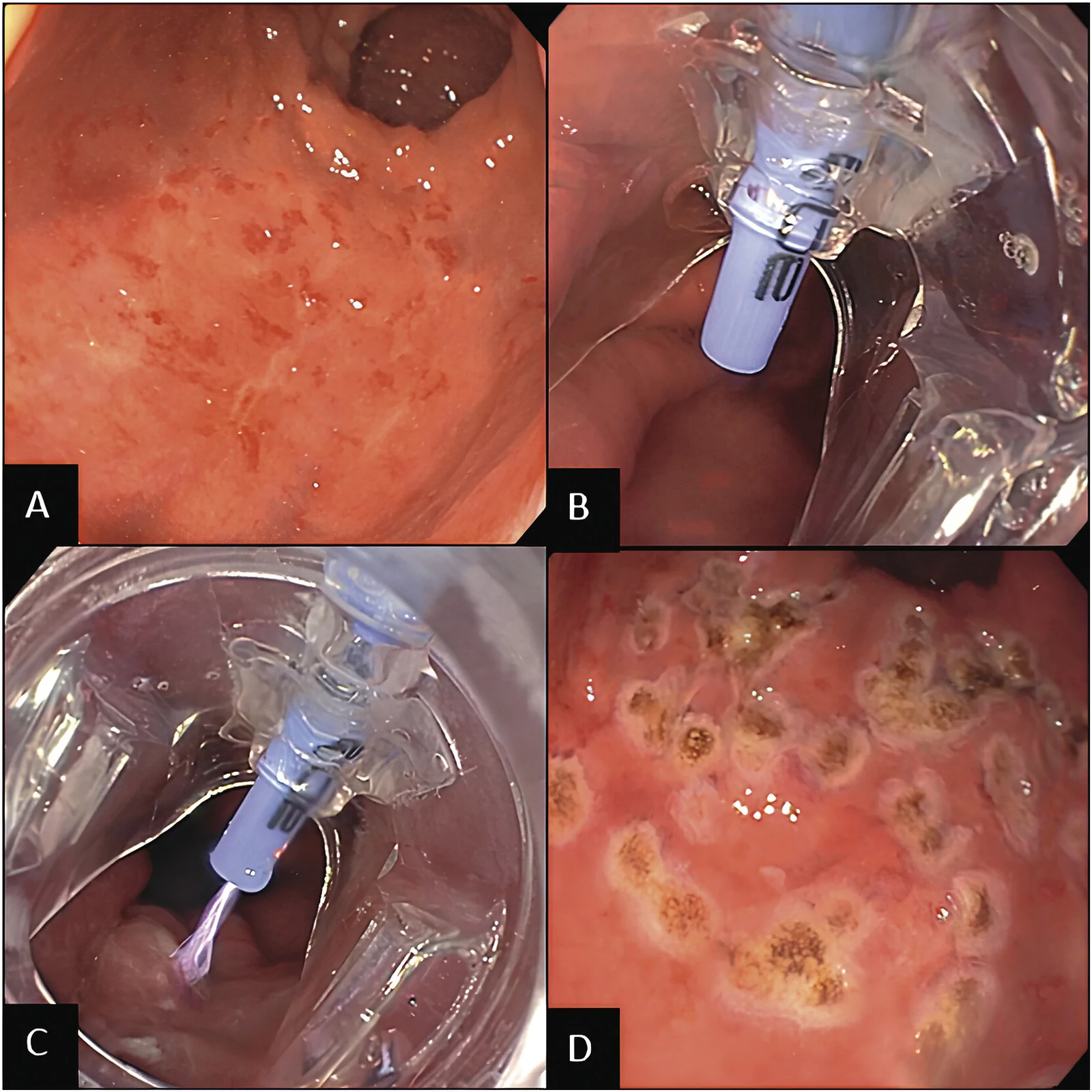
Treatment of radiation proctitis. ( **A** ) Diffuse radiation proctitis, ( **B** ) Endoscopic view with ArgoCap mounted, ( **C** ) APC therapy, ( **D** ) Post-treatment result.

**Fig. 3:**
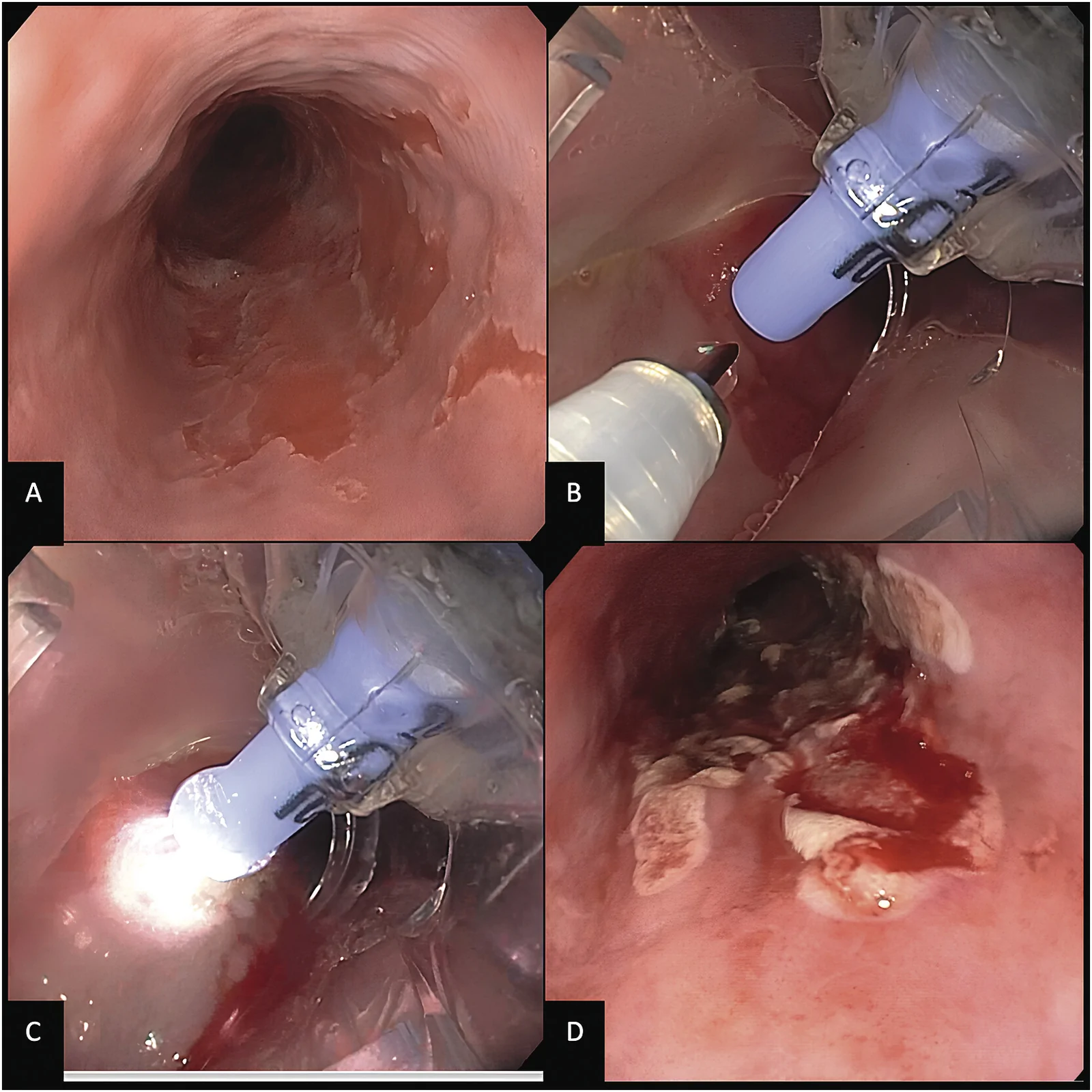
Hybrid APC treatment of Barrett´s esophagus. ( **A** ) Barrett esophagus C8M9, ( **B** ) Endoscopic view with ArgoCap mounted and injection needle through working channel, ( **C** ) APC therapy, ( **D** ) Post-treatment result.

A total of 54 applications of the ArgoCap were analyzed ( Table 1 ). Mean age of the cohort at the time of intervention was 62 (±16) years, with a ratio of men to women of 2:1. Endoscopically treated lesions included Barrett’s mucosa (20, 37%), ectopic gastric mucosa (15, 28%), gastric antral vascular ectasia (GAVE, 6, 11%), radiation proctitis (6, 11%), and other indications (7, 13%; *n* = 2 anastomotic treatments after bariatric outlet reduction, *n* = 2 preconditioning of an esophageal lymph fistula, *n* = 1 bleeding of a gastric corpus gland cyst, *n* = 1 gastric angiodysplasia, *n* = 1 ablation of an esophageal stent cover) ( Figs. 2 and 3 ). Most of the target lesions were located in the esophagus (70%), followed by gastric (19%) and rectal lesions (11%). The median maximal lesion size was 13 mm (range 2–240 mm) including an extensive Barrett’s esophagus measuring 240 mm significantly larger than the other lesions. There are no precise data available on the lesion sizes documented as extensive in cases of GAVE and radiation proctitis, as well as in other indications. Hybrid APC with submucosal injection before ablation was conducted in 4 cases (7%): In two extensive Barrett lesions (C5M7 and C8M9 according to Prague classification) and the two cases of esophageal lymph fistula. APC modes and power settings are listed in Table 1 . There was no significant difference in power settings among rectal, gastric, and esophageal lesions ( *p* = 0.64). The median ablation time was 8 min (range 2–15 min), with a total procedure duration of 25 min (range 8–75 min). There was no significant difference regarding the ablation time between the different indications ( *p* = 0.85). Overall, 96% of the lesions could be reached endoscopically; in 2 (33%) cases of GAVE not 100% could be reached. Overall, 4% of the cases were treated in retroflexion, and in these cases all target structures were reached. Technical success, defined as access to the target lesion and macroscopically complete ablation, was overall achieved in 47 cases (87%), respectively. The incompletely treated lesions consisted of five cases of GAVE without further information about the lesion size, one case of Barret’s mucosa of extensive size (C24M24 according to Prague classification), and one case of radiation proctitis. However, in 4 of these 7 cases, a subtotal ablation of >90% of the surface could be achieved. For the incompletely treated lesions, a further conventional APC treatment was successfully carried out without the ArgoCap. Technical difficulties occurred in 1 case (2%), in which the taped cap slipped during insertion from the endoscope into the esophagus. A procedure-related moderate adverse event was reported in *n* = 1 case (2%), where treatment of GAVE led to ulceration with subsequent bleeding. The bleeding could be successfully treated endoscopically.

**Table 1:** Baseline characteristics and results.

	Overall *n* = 54 (100)	Barrett mucosa *n* = 20 (37)	Ectopic gastric mucosa *n* = 15 (28)	GAVE *n* = 6 (11)	Radiation proctitis *n* = 6 (11)	Other indications *n* = 7 (13)
Age, *years (SD)*	62 (16)	62 (16)	60 (17)	69 (11)	64 (15)	66 (12)
Sex, *n (%)*						
male	36 (66)	19 (95)	6 (40)	3 (50)	5 (83)	3 (43)
female	18 (33)	1(5)	9 (60)	3 (50)	1 (17)	4 (57)
Localization, *n (%)*						
Esophagus	38 (70)	20 (100)	15 (100)	–	–	3 (43)
Stomach	10 (19)	–	–	6 (100)	–	4 (57)
Rectum	6 (11)	–	–	–	6 (100)	–
Max. lesion size, *median (range) mm*	13 (2–240)	13 (2–240)	13 (2–30)	n.a.	n.a.	n.a.
Hybrid APC, *n (%)*	4 (7)	2 (10)	–	–	–	2 (29)
APC Mode, *n (%)*						
Precise	20 (37)	16 (80)	1 (7)	2 (33)	–	1 (14)
Pulsed	30 (55)	4 (20)	14 (93)	3 (50)	5 (83)	4 (57)
Forced	4 (8)	–	–	1 (17)	1 (17)	2 (29)
Power setting, *n (%)*						
11–16 W	27 (50)	10 (50)	10 (66)	3 (50)	4 (66)	–
17–21 W	17 (31)	9 (45)	4 (27)	2 (34)	1 (17)	1 (14)
>21 W	10 (19)	1 (5)	1 (7)	1 (17)	1 (17)	6 (86)
Procedural time, *median (range) min*	25 (8–75)	25 (8–39)	26 (14–73)	25 (18–70)	25 (15–46)	25 (15–75)
Ablation time	8 (2–15)	8 (2–15)	8 (2–15)	8 (7–15)	10 (5–15)	8 (5–10)
Target lesion reached, *n (%)*	52 (96)	20 (100)	15 (100)	4 (66)	6 (100)	7 (100)
Complete ablation, *n (%)*	47 (87)	19 (95)	15 (100)	1 (17)	5 (83)	7 (100)
> 90 % ablation, *n (%)*	52 (96)	19 (95)	15 (100)	5 (83)	6 (100)	7 (100)
≤ 90% ablation, *n (%)*	2 (4)	1 (5)	–	1 (16)	–	–
Technical complications, *n (%)*	1 (2)	1 (5)	–	–	–	–
Adverse events, *n (%)*	1 (2)	–	–	1 (17)	–	–

## Discussion

Argon plasma coagulation (APC) is a well-established, effective, and safe thermal treatment technique in endoscopy with a wide range of applications. However, its use is limited by the solely tangential orientation of the probe causing collateral damage to adjacent mucosa or resulting in unintended mucosal contact. To overcome this limitation and facilitate APC treatment, the ArgoCap was developed. In the present study, the use of ArgoCap was evaluated for various indications in the upper and lower gastrointestinal tract.

The primary endpoint, i.e., technical success, was achieved in 87% of cases. Among the seven incompletely ablated lesions were large-area- ablations (five GAVE cases, one extensive Barrett’s case (C24M24), and one case of radiation proctitis), generally known to be complex for single APC-therapy. In general, extensive Barrett’s lesions are not typical for endoscopic APC treatment alone; the decision for APC treatment was made on a case-by-case decision here. Previous studies on lesions with a median size of 4 cm have been reported. 7 10 Excluding this large Barrett’s case, the corrected technical success rate was 90%. In radiation proctitis, a median of two sessions is also typically required to control rectal bleeding. 2 3 The maximum lesion size was likely underestimated due to missing data for GAVE and radiation proctitis. Although accessibility is less compromised than in the stomach, these lesions are often extensive and poorly demarcated. In 96% of cases, the target lesion could be reached endoscopically. In the esophagus and rectum, all lesions were accessible. Only in the stomach—specifically in cases of GAVE— complete access to all affected areas achieved in only two-thirds of the procedures. Areas that are difficult to reach endoscopically, such as the gastric fundus and the cardia region, in inverted position might be the cause. APC treatment of GAVE typically requires multiple sessions and is associated with a high recurrence rate of up to 40%. 11 12 The usually diffuse and extensive distribution of lesions, the gastric folds, and the challenging orientation within the large gastric cavity likely contribute to the reduced endoscopic accessibility and complete ablation. A direct comparison with success rates of conventional APC therapy is not meaningful, as no follow-up examinations were available in this study. The procedure time for esophageal applications was comparable to published results. 10 The quick assembly and disassembly of the ArgoCap system allowed peri-interventional switching between different techniques. Technical difficulties occurred in only one case, in which the cap dislodged while being introduced into the esophagus. Proper fixation of the cap using the supplied adhesive strips on a dry endoscope is essential to ensure a secure fit.

In the present study, only one moderate adverse event (2%) occurred using the ArgoCap. They are in line with the reported complication rates for APC therapy reported in literature and vary between 1.4-6% considerably depending on indication and location and are mainly related to deep tissue injury caused by excessive power settings or prolonged application. 11 13 14 15 16 17 In our study, the power settings were substantially lower than those typically reported in the literature. This was based on findings from our prior ex vivo experiments, which demonstrated that angled APC application requires less energy for precise therapy. 9 Moreover, the open working channel of the endoscope allows even hybrid APC therapy, enabling more tissue-sparing ablation. Especially in esophageal applications, this approach may help reduce the risk of postinterventional stenosis. 7 Recently, Erbe introduced the FiAPC 2200 A probe, a side-firing APC catheter that redirects the plasma beam by 90° and can be used via the working channel. Although robust clinical data on this system are still lacking, the ArgoCap offers several distinct advantages, such as improved visibility of the APC beam, more precise ablation, protection of adjacent mucosal areas and prevention of beam scattering, allowing a predetermined distance between the tip and tissue, minimizing deep thermal injury, and enabling the possibility of suction and hybrid-APC application, as the working channel remains free.

Additionally, the shape of the cap allows for gentle tissue tensioning and improved visualization, particularly useful in narrow luminal areas. These advantages are especially evident during intraluminal esophageal applications, which we consider the primary field of use for the ArgoCap system. These advantages are intended to make APC treatment easier, particularly for hard-to-reach lesions or inexperienced endoscopists. However, when considering esophageal APC therapy for Barrett’s esophagus, it is important to note the limited level of evidence supporting the therapy, the risk of glandular burns, and the potential risk of submucosal fibrosis, which can complicate further endoscopic treatment. Limitations of this study include its retrospective design and the heterogeneous patient cohort without predefined indications or power settings. Additionally, a direct comparison with success rates of conventional APC therapy is not meaningful, as no follow-up examinations were available in this study.

In summary, the study highlights the ease of use and wide range of applications of ArgoCap. Further prospective studies are warranted to validate these results and to assess potential clinical advantages of the device.
